# Navigating Treatment Sequencing in Advanced HR+/HER2− Breast Cancer After CDK4/6 Inhibitors: Biomarker-Driven Strategies and Emerging Therapies

**DOI:** 10.3390/ijms262110366

**Published:** 2025-10-24

**Authors:** Dana P. Narvaez, David W. Cescon

**Affiliations:** Department of Medical Oncology and Hematology, Princess Margaret Cancer Centre, University Health Network, Toronto, ON M5G 2C4, Canada; narvaezdanap@gmail.com

**Keywords:** metastatic breast cancer, luminal, hormonal resistance, cyclin-dependent kinase 4/6 inhibitor

## Abstract

Breast cancer remains a major global health challenge. In 2022, there were an estimated 2.3 million new cases and 670,000 deaths among women worldwide. Hormone receptor-positive (HR+)/human epidermal growth factor receptor 2-negative (HER2−) breast cancer accounts for approximately 70% of breast cancer diagnoses. The treatment landscape for advanced HR+)/HER2− breast cancer has been transformed by the introduction of CDK4/6 inhibitors in the first-line setting. However, therapeutic strategies following progression on CDK4/6 inhibitors remain heterogeneous and uncertainty exists in their optimal integration in clinical practice. This review aims to systematically examine available second-line and subsequent treatment options for HR+/HER2− metastatic breast cancer after progression on CDK4/6 inhibitors, with a focus on biomarker-driven strategies and emerging therapies. The therapeutic landscape beyond CDK4/6 inhibitors includes targeted agents guided by actionable biomarkers as well as novel selective estrogen receptor degraders (SERDs). In biomarker-unselected populations, options include CDK4/6 continuation strategies, endocrine monotherapy in selected cases, and cytotoxic therapy. The integration of molecular testing via next-generation sequencing has become standard of care in guiding these decisions. However, overlapping molecular alterations and a lack of consensus on treatment sequencing pose significant challenges. Prognostic factors such as circulating tumor DNA dynamics may further refine treatment personalization. Post-CDK4/6 therapy in HR+/HER2− metastatic breast cancer is an evolving and increasingly complex area of practice. Optimal treatment selection should be tailored to both tumor biology and patient-specific factors, supported by molecular testing and high-quality evidence.

## 1. Introduction

Breast cancer (BC) is a significant global health challenge, with regional disparities in outcomes reflecting differences in access to care and advances in treatment [1]. In North America, while breast cancer incidence has steadily increased over the last 4 decades, survival rates have improved, largely due to advancements in early detection and systemic therapies [2]. However, metastatic breast cancer, regardless of geographic region, remains incurable, highlighting a continued need for new and improved treatment strategies. In 2022, there were an estimated 2.3 million new cases and 670,000 deaths among women worldwide. By 2050, the number of new cases and deaths is projected to rise by 38% and 68%, respectively [3]. This review focuses on the evolving therapeutic landscape for advanced HR+/HER2-negative breast cancer, with specific attention to treatments following disease progression on standard first-line therapy with CDK4/6 inhibitors.

Hormone receptor-positive (HR+)/human epidermal growth factor receptor 2-negative (HER2−) breast cancer accounts for approximately 70% of breast cancer diagnoses [4].

Advanced breast cancer is an incurable disease, and treatment goals are recognized to encompass not only efforts to improve patient survival but also to maintain and optimize quality of life and symptom control, and take into consideration patient preferences and other individual factors [5]. Current guidelines recommend the combination of endocrine treatment (aromatase inhibitor or fulvestrant) and a cyclin-dependent kinase 4/6 inhibitor (CDK4/6i) as first-line treatment for advanced HR+/HER2− breast cancer [6]. The introduction and widespread incorporation of CDK4/6 inhibitors in combination with endocrine therapy since 2015 have changed the treatment landscape and improved outcomes [7].

The three CDK4/6i available globally have demonstrated improvements in progression-free survival (PFS) when added to aromatase inhibitors and fulvestrant, with ribociclib also showing a significant improvement in overall survival (OS) in the registrational trials [8,9,10,11]. These efficacy data, combined with their tolerability profile and supportive quality of life data, have firmly established this first-line approach. However, optimal treatment after progression on a CDK4/6 inhibitor has not been standardized in the same way, with several treatment strategies being developed in parallel. Our understanding of the efficacy of post-CDK4/6i therapy comes from retrospective and real-world studies as well as an increasing number of prospective trials that have been completed and reported in this population [12,13,14]. Selecting optimal second and third-line therapies for patients with HR+/HER2− metastatic breast cancer requires careful consideration of disease characteristics (tumor biology, hormonal resistance, and presence or absence of predictive biomarkers) and patient characteristics (symptoms and disease burden, treatment toxicities, frailty, performance status, and patient preferences).

There is currently no single predictive biomarker in clinical practice that allows for optimal treatment sequence selection [6]. Moreover, second-line treatment options for HR-positive and HER2-negative metastatic breast cancer patients have increased with the approval of new agents and the evaluation of existing agents in patients previously treated with first-line CDK4/6i. In some cases, treatment strategies are linked to actionable biomarkers, consisting of somatic or germline mutations [5]. The first-line AI+CDK4/6i regimens are summarized in Table 1 (see Table 1).

The latest updates of international recommendations for HR+/HER2-negative metastatic breast cancer (MBC) suggest next-generation sequencing of a tumor sample (or plasma circulating tumor DNA, ctDNA) as a standard of care to search for mutations that can be actionable with targeted therapies [16]. These include key genomic alterations such as PIK3CA, ESR1, and germline *BRCA1/2* mutations, which have been incorporated as biomarkers in clinical trials (see Table 2).

The objective of this review is to systematically outline treatment options available or under investigation following progression on CDK4/6 inhibitor therapy. We aim to present both biomarker-selected and unselected approaches, highlighting patient characteristics included in clinical trials. In addition, we will examine study results with a focus on primary clinical endpoints and consider the appropriate timing and method for molecular testing. The goal is to summarize the role of a ‘biomarker-first’ approach, in which predictive tools such as ctDNA guide treatment selection for each individual, and define the therapeutic landscape through which ongoing clinical trials will be interpreted.

A systematic search was conducted in PubMed, Embase, and ClinicalTrials.gov from 1 July 2004, to 30 August 2025, to identify relevant clinical trials addressing post-CDK4/6 inhibitor treatments in patients with hormone receptor positive (HR+) and human epidermal growth factor receptor 2-negative (HER−) advanced breast cancer. In addition, the authors reviewed retrospective studies, meta-analyses, narrative reviews, and preclinical studies related to treatment sequencing after progression on first-line CDK4/6 inhibitors. The search terms included “CDK4/6 inhibitor”, “second-line treatment”, “biomarker”, “progression” “ctDNA” and “targeted treatment” to ensure specificity. Articles were selected by the authors based on their relevance to the development of this narrative review.

## 2. Results

### 2.1. Natural History of HR-Positive/HER2-Negative Breast Cancer: Endocrine Resistance and CDK4/6 Inhibitors Resistance

Primary endocrine resistance, a clinical definition, has been defined as relapse within the first 2 years of adjuvant endocrine therapy or disease progression within the first 6 months of first-line endocrine therapy for advanced or MBC. Secondary resistance in early breast cancer is defined as relapse occurring after at least 2 years of endocrine therapy and during or within the first year after completing adjuvant endocrine therapy. On the other hand, secondary resistance in MBC is defined as disease progression after more than 6 months of endocrine therapy [27]. These definitions therefore capture different clinical scenarios, with potentially different biology of resistant disease, under a common umbrella.

Approximately 10% of patients will have primary resistance to CDK4/6 inhibitors in the metastatic setting [28]. Preclinical and translational research studies have attempted to characterize the genomic and/or molecular basis of this resistance, and various factors have been identified to contribute to this phenomenon in the first line of treatment. A simple way to classify these alterations is to divide them into those affecting cell cycle mediators and those involving the activation of oncogenic signal transduction pathways [29].

Several biological parameters are associated with prognosis on CDK4/6i-based therapy. Witkiewicz and colleagues analyzed a cohort of 280 patients who received palbociclib and observed that shorter PFS was statistically associated with microscopic grade (modified Scarff–Bloom–Richardson (SBR) scores) and PR immunohistochemistry levels (HR = 3.86, *p* = 0.008). [30] According to the results of AURORA, which analyzed 339 patients treated with CDK4/6 inhibitors plus endocrine therapy in the first line, the association with poorer PFS varied significantly between endocrine resistance subgroups (*p* < 0.001). Plasma analyses were performed through serial sampling: blood samples were collected routinely every six months and at the time of disease progression. In a multivariable analysis, acquired mutations in *ESR1* were significantly associated with worse PFS regardless of endocrine resistance status (HR 2.42 *p* = 0.048) [31].

When evaluated in pivotal studies, such as MONALEESA-2, the treatment benefit of CDK4/6i was maintained regardless of the mutational status of any of the tested biomarker alterations, including *ESR1* [32]. In MONARCH 3, *ESR1* mutations were infrequent at baseline but were associated with numerically shorter median PFS in both treatment arms. Acquired *ESR1* alterations at the time of treatment failure were observed in 20% of patients in the abemaciclib arm and 32% in the placebo arm [11]. In the MONARCH 2 study population, benefit of abemaciclib was observed in both the baseline *ESR1* wild-type subgroup (median PFS: 15.3 months vs. 11.2 months; HR: 0.44) and the *ESR1*-mutant subgroup (median PFS: 20.7 months vs. 13.1 months; HR: 0.54). Improvement in efficacy endpoints with abemaciclib, including OS, were independent of PIK3CA or *ESR1* mutation status [33,34].

Altogether, these findings support the current consensus that CDK4/6i in the first-line setting improve outcomes when added to endocrine therapy, irrespective of other clinical or molecular features. Analyses of patients treated in the first-line setting, including the frequent emergence of acquired ESR1 mutations, provide evidence that such alterations can be detected as they arise and may inform new treatment approaches. This strategy has shown promise in adaptive trials such as SERENA-06 and PADA-1 where real time *ESR1* detection was used to guide early therapeutic intervention (discussed further below) [24,35].

### 2.2. Second-Line Biomarker-Based Therapies

Several biomarker-based treatment strategies exist for second-line therapy. Some relevant biomarkers arise as acquired somatic alterations that can occur during first-line therapy (e.g., *ESR1*), while others are common truncal mutations typically present at diagnosis (e.g., PIK3CA) [36,37]. Certain biomarker-based therapies were developed prior to the availability or widespread use of CDK4/6i in the first-line setting, or were evaluated in studies that enrolled global populations where receipt of first-line CDK4/6i was low. It has been recognized that outcomes on at least some second-line endocrine-based therapies may differ based on prior receipt of CDK4/6i. Therefore, the characteristics of enrolled populations must be considered when applying trial evidence to patients who now nearly universally receive CDK4/6i in settings where resources permit.

Table 3 summarizes clinical trials with drugs offering biomarker-guided treatment options, including both FDA-approved and non-approved therapies, along with a description of study design and outcomes.

#### 2.2.1. PI3K Pathway: PI3K and AKT Inhibitors

The PI3K pathway is a key oncogenic signaling process in HR+ breast cancer. The clinical development of inhibitors of this pathway, focused on patients with somatic alterations that activate it, has been underway for a decade and a half. Mutations in PIK3CA are found in 35–40% of mBC, and most commonly in HR+ disease [38].

The first drug approved for the treatment of breast cancer associated with PI3K mutations was alpelisib. In the phase III SOLAR-1 trial, alpelisib combined with fulvestrant demonstrated a statistically significant prolongation of PFS, but this did not translate into a significant improvement in OS (a numerical gain of 8 months in the final analysis) [17]. Among patients with *PIK3CA*-mutated disease as detected using plasma ctDNA, the median OS was 34.4 months in the alpelisib plus fulvestrant arm, compared with 25.2 months (HR = 0.74) [17]. Prior use of CDK4/6i in SOLAR-1 was rare, due to the timing of the study’s enrollment. To evaluate the activity of alpelisib in patients who had received CDK4/6i inhibitors in the first line, the non-randomized phase II BYLIEVE trial was conducted. In this study, alpelisib in combination with endocrine therapy demonstrated a median OS ranging from 20.7 months to 29.0 months following prior CDK4/6 inhibitors, chemotherapy, or endocrine therapy [39]. The overall response rate among patients with measurable disease at baseline in BYLieve (21%) was lower than that observed in SOLAR-1 (35.7%). To definitely address the role of alpelisib in combination with fulvestrant in the post-CDK4/6i setting, a phase III trial in this population (EPIK-B5) is underway. Accrual has been completed, with primary results expected in 2026 [40].

CapItello-291, which evaluated the AKT inhibitor capivasertib in combination with fulvestrant, is the second phase III trial of an agent targeting this pathway that led to clinical approval based on PFS improvement in a biomarker-defined subset of patients with AKT pathway (*PIK3CA, AKT, PTEN*) alterations. Of the 602 participants with tumor sequencing results, 48.0% had AKT pathway alterations, most commonly PIK3CA mutations, while mutations in more than one gene were rare. In the AKT pathway-altered population (as determined in archival tissue), the median PFS was 7.3 months in the capivasertib–fulvestrant group versus 3.1 months in the placebo–fulvestrant group (HR, 0.50; 95% CI, 0.38–0.65; *p* < 0.001). Among the 489 (69.1%) patients who had received prior CDK4/6 inhibitors with an aromatase inhibitor, those treated with the doublet (*n* = 248) had a median PFS of 5.5 months (95% CI, 3.9–6.8) versus 2.6 months (95% CI, 2.0–3.5) with fulvestrant alone (adjusted HR, 0.59; 95% CI, 0.48–0.72). In contrast, among patients without prior CDK4/6i exposure, the median PFS was 10.9 months with the combination and 7.2 months with fulvestrant alone (HR, 0.64).

Results were also reported in the AKT-non altered population: when including those with unknown NGS status (*n* = 419), the HR for progression or death was 0.70 (95% CI, 0.56–0.88); when excluding patients with unknown NGS results (*n* = 313), the HR was 0.79 (95% CI, 0.61–1.02); and among patients with unknown results (*n* = 106), the HR in post hoc analysis was 0.52 (95% CI, 0.32–0.83) [18]. On the basis of these results, the use of capivasertib has been limited to patients with AKT pathway alterations, where the treatment effect is greatest.

No trial has directly compared the efficacy of capivasertib and alpelisib in combination with fulvestrant, and the differences in the biomarker selection for these therapies (PIK3CA only, vs. three AKT pathway genes) must be noted. The median PFS observed in the overall population of the CAPItello-291 study with capivasertib plus fulvestrant is similar to that reported in the phase II BYLieve trial.

At ASCO 2025, the results of the CCTG MA.40/FINER trial, which evaluated the addition of the AKT inhibitor ipatasertib to fulvestrant in the second-line post-CDK4/6i setting, were presented. This phase 3 trial required that all enrolled patients had previously received and had disease progression on a CDK4/6i and aromatase inhibitor as the immediate prior line of therapy, and stratified participants based on AKT pathway alterations identified by ctDNA liquid biopsy at the time of enrolment. The primary results showed a median PFS in the ITT population for the ipatasertib vs. placebo arms of 5.32 months vs. 1.94 months (HR 0.61; 95% CI: 0.46–0.81; *p* = 0.0007). In the AKT pathway-altered cohort, median PFS was 5.45 months vs. 1.91 months (HR 0.47; 95% CI: 0.31–0.72; *p* = 0.0005) [19]. These results support the role of AKT targeting in patients with AKT pathway-altered tumors in the second-line setting, and also provide additional data on the very limited efficacy of fulvestrant monotherapy for most patients in the post-CDK4/6i setting.

Across trials, AKT and PI3K pathway inhibitors were generally associated with manageable but notable toxicity profiles, with some variability in severity and discontinuation rates. In the FINER study, grade ≥3 adverse events occurred in 37.1% of patients treated with ipatasertib plus fulvestrant, most commonly diarrhea (16%), fatigue (3%), vomiting (2%), and rash (2%), with a relatively low treatment discontinuation rate of 6.5%. In CAPItello-291, capivasertib plus fulvestrant led to grade ≥3 rash in 12.1% and diarrhea in 9.3% of patients, with a discontinuation rate of 13.0%, notably higher than 2.3% in the placebo group. In BYLieve, which investigated alpelisib plus fulvestrant, the most frequent grade ≥3 adverse events included hyperglycemia (29%), rash (10%), and maculopapular rash (9%), with 29% experiencing serious adverse events, although no treatment-related deaths were reported. Overall, while these targeted combinations can offer clinical benefit, their distinct toxicity profiles (particularly dermatologic, gastrointestinal, and metabolic) necessitate careful patient selection, proactive monitoring, and supportive care to maintain treatment adherence and quality of life [19,20,21].

#### 2.2.2. Targeting ESR1 Mutations: Novel SERDS

Fulvestrant, a non-orally bioavailable SERD, was approved in the early 2000s after demonstrating outcomes comparable to anastrozole in advanced disease [41]. A new generation of oral Selective Estrogen Receptor Degraders (SERDs) has emerged as a promising class of endocrine therapy, particularly in the setting of aromatase inhibitor resistance driven by *ESR1* mutations. Various SERD-based combination strategies are under investigation to enhance efficacy and delay progression in HR+/HER2− advanced breast cancer after CDK4/6 inhibitor and aromatase inhibitor treatment failure. Selective estrogen receptor degraders (SERDs) bind to the estrogen receptor, promoting its degradation and inhibiting intracellular signaling [42,43]. The clinical landscape of oral SERDS and related therapies is poised to expand, after the initial approval of elacestrant monotherapy, based on multiple clinical trials in various combinations and settings.

Elacestrant was the first new endocrine therapy to receive FDA approval since fulvestrant, based on the EMERALD study. EMERALD enrolled participants with ER-positive/HER2-negative advanced breast cancer who had received one to two prior lines of endocrine therapy, mandatory pretreatment with a CDK4/6 inhibitor, and no more than one line of chemotherapy. In the subgroup of patients with detectable ESR1 mutations in ctDNA, elacestrant demonstrated a statistically significant improvement in median PFS (5.5 vs. 3.3 months) compared to standard endocrine monotherapy (HR = 0.55; 95% CI: 0.39–0.77; *p* = 0.0005). [34] No benefit was observed in the non-ESR1 mutation population, with similar PFS as SOC-ET [21].

Given the short PFS and small absolute PFS improvement observed in the overall study population, several clinical subgroups have been evaluated in EMERALD, in an effort to identify patients most likely to have more durable disease control. Duration of prior therapy in the 1L setting has been shown to correlate with PFS on elacestrant: in patients with prior exposure to ET + CDK4/6i for ≥12 months and *ESR1*-mutated tumors, treatment with elacestrant resulted in an mPFS of 8.6 months vs. 1.9 months with standard-of-care endocrine therapy (HR = 0.41; 95% CI: 0.26–0.63).

ESR1 mutations are typically subclonal. In addition, variant allele fraction (VAF) in ctDNA correlates with tumor burden and could serve as a prognostic or predictive biomarker. A post hoc analysis of EMERALD was conducted in patients with *ESR1*-mutated, endocrine-sensitive tumors (prior ET + CDK4/6i exposure for ≥12 months). The median ESR1 mutant variant allele frequency was 1.2% (95% CI: 0.04–56.1%). VAF levels were accordingly categorized as high (≥1.2%) and low (<1.2%). The D538G, Y537S, and Y537N variants accounted for nearly 90% of detected *ESR1* mutations. Elacestrant was associated with a PFS improvement across all *ESR1* mutation variants. Notably, the VAF level (high vs. low) did not impact the PFS benefit associated with elacestrant compared to SOC. On the other hand, in the presence of coexisting *ESR1* and *PIK3CA* mutations, a greater PFS improvement with elacestrant vs. standard-of-care endocrine therapy was observed. This benefit was evident despite 89% of these patients having a lower *ESR1* variant allele frequency compared to *PIK3CA* VAF (consistent with their subclonal/acquired and truncal natures) [44].

These findings contribute to a better understanding of elacestrant’s clinical activity and its role as monotherapy in the management of *ESR1*-mutated advanced breast cancer. However, uncertainties remain about which treatment sequencing strategy is optimal for patients where multiple options may exist (e.g., *PIK3CA/AKT* and *ESR1* co-mutated tumors)—a very common occurrence.

In the MA.40/FINER population, which included patients enrolled following standard first-line therapy with an aromatase inhibitor and CDK4/6 inhibitor, exploratory analyses of PFS by ESR1 mutation status were reported in the ASCO 2025 presentation. Among patients with ESR1 wild-type tumors (*n* = 128; 51%), median PFS was 5.55 months (95% CI: 3.71–12.88) in the ipatasertib arm versus 2.00 months (95% CI: 1.84–3.68) in the placebo arm, with a hazard ratio (HR) of 0.54 (95% CI: 0.36–0.83). In the ESR1-mutant cohort (*n* = 122; 49%), median PFS was 3.71 months (95% CI: 2.04–5.45) with ipatasertib compared to 1.87 months (95% CI: 1.77–4.24) with placebo, yielding an HR of 0.61 (95% CI: 0.41–0.91) [20].

An unanswered question is whether the use of elacestrant in combination with other targeted treatments will provide additional efficacy. The phase 1b/2 ELEVATE trial aims to determine the recommended phase 2 dose (RP2D) of elacestrant when administered in combination with alpelisib, everolimus, palbociclib, abemaciclib, and ribociclib. The phase 2 part of the trial will evaluate the efficacy and safety of these various combinations in patients with HR+/HER2− advanced/metastatic breast cancer [45].

Several phase 2 and 3 trials of oral SERD and related agent (e.g., SERCAs, PROTACs) monotherapy have been conducted in this setting, including SERENA-2 (camizestrant), acelERA (giredestrant), and VERITAC (vepdegestrant). While the patient populations have slightly different characteristics (including the degree of prior CDK4/6i use), the results of these trials have been similar to EMERALD, with modest absolute improvements in PFS compared to fulvestrant, enriched in the population with detectable *ESR1* mutations [46,47,48].

**Table 3:** Biomarker-Guided Treatment.

Drug	Year	Biomarker	Design	*n*	Patient Characteristics	Previous Treatment Allowed	ORR	PFS	OS	QoL	Toxicity Profile	FDA Approved (August 2025)
SOLAR-1 Alpelisib plus fulvestrant (NCT02437318) [17]	2019	PIK3CA (PCR on tumor tissue)	Phase III	572	341 patients with PIK3CA mutations (59.6%) and 231 patients with PIK3CA wild-type tumors	Disease progression on or after AI	26.6%	Median PFS 11.0 months (HR 0.65 *p* = 0.00065).	Median OS 39.3 vs. 31.4 months (HR) = 0.86 (95% CI, 0.64 to 1.15; *p* = 0.15).	Maintained QoL and functioning	Grade 3 or 4 hyperglycemia 36.6% and G3 or G4 rash 9.9%. Grade 3 diarrhea in 6.7%. Discontinuation rate 25.0%	Yes
BYLieve Alpelisib plus fulvestrant (NCT03056755) [40]	2021	PIK3CA (NGS and PCR on tumor tissues)	Phase II	127	121 patients with centrally confirmed PIK3CA mutation	Progression on or after previous therapy, including CDK4/6i.	17%	Median PFS 7.3 months	Median OS 17.3 months (95% CI 17.2 to 20.7)	Did not adversely affect overall health-related quality of life	Grade 3 hyperglycaemia 28%, G3 rash 9% [32]	Yes
CAPITELLO-291 (*) Capivasertib plus fulvestrant (NCT04862663) [19]	2023	PIK3CA (tumor tissue NGS using the FoundationOne CDx assay)	Phase III, randomized, double-blind trial	708	289 patients (40.8%) with AKT pathway alterations, and 489 (69.1%) had received a CDK4/6i for advanced breast cancer	Disease progression on AI, with or without CDK4/i. Up to 2 prior lines of ET and 1 prior line of chemotherapy	28.8% in the biomarker-altered population	Median PFS 7.3 months (HR 0.50 *p* < 0.001)	OS at 18 months 73.2% (95% CI, 64.8 to 80.0) vs. 62.9% (95% CI, 53.1 to 71.2) in the AKT pathway-altered population (HR, 0.69; 95% CI, 0.45 to 1.05)	Global health status and quality of life were maintained in both arms (QLQ-C30)	Grade 3 rash 12.1% and Grade 3 diarrhea 9.3%. Discontinuation rate of 13.0%	Yes
FINER Ipatasertib (NCT04650581) [20]	2025	AKT pathway-altered (PIK3CA, AKT1, and/or PTEN) in ctDNA at enrolment (FoundationOne Liquid)	Phase III, randomized, double-blind trial	250	44.4% of the study population had AKT pathway alteration(s)	Disease progression on 1L AI+CDK4/6i as immediate prior therapy	N/R	Median PFS 5.3 vs 1.9 months (ITT) (HR 0.61 *p* = 0.0007)	N/R	N/R	Grade 3 or higher AEs for ipatasertib 37.1%. Discontinuation rate 6.5%	No
EMERALD (***) Elacestrant (NCT03778931) [21]	2022	ESR1 (ctDNA analysis using the Guardant360 assay)	Randomized, open-label, phase III trial	477	*ESR1* mutation was detected in 47.8% of patients	1–2 prior lines of ET. Prior CDK4/6i required, ≤ 1 chemotherapy.	15.2%	In patients with ESR1^mut^ and prior ET+CDK4/6i ≥12 months, median PFS 8.6 months (HR 0.41)	OS (interin analysis) HR of 0.75 (95% CI, 0.54 to 1.04; *p* 0.08). In patients with ESR1 mutation HR of 0.59 (*p* 0.03 non-significant)	Maintaining QOL	Grade 3/4 AEs occurred in 7.2% Discontinuation rate 3.4%	Yes
VERITAC-2 Vepdegestrant (NCT05654623) [23]	2025	ESR1 (ctDNA testing by Foundation Medicine, except in China where Origmed testing was used)	Phase 3, open-label, randomized trial	624	*ESR1* mutation was detected in 270 patients (43.3%)	1 line of CDK4/6i plus 1 line of ET	18.6%	In patients with ESR1 mutations median PFS was 5.0 months (HR 0.58 *p* < 0.001). ITT population median PFS was 3.8 months (HR 0.83 *p* = 0.07)	N/R	N/R	Grade 3/4 AEs occurred in 23.4%. Discontinuation rate 2.9%	No
SERENA 2 (**) Camizestrant (NCT04214288) [47]	2022	ESR1 mutation in ctDNA at enrolment by NGS	Phase II	240	*ESR1* mutation was detected in 36.7% of patients	Recurrence or progression on at least 1 line of ET. ≤1 line of ET for ABC. ≤ 1 chemotherapy for ABC No prior fulvestrant	15.7% with 75 mg and 20.3% with 150 mg	Median PFS 7.2 months (camizestrant 75 mg) HR 0.58 *p* = 0.0124 and 7.7 months (camizestrant 150 mg) HR 0.67 *p* = 0.0161	N/R	N/R	AEs leading to treatment discontinuation occurred in 2.7%	No
EMBER-3 imlunestrant (NCT04975308) [22]	2024	ESR1 mutation in ctDNA at enrolment	Phase 3, open-label trial	874	*ESR1* mutation detected in 256 patients (29.3%)	Recurred or progressed during or after AI, alone or with a CDK4/6i	27% with the imlunestrant–abemaciclib combination and 12% with imlunestrant monotherapy	In patients with ESR1 mutations, median PFS 5.5 (imlunestrant) (*p* < 0.001) 9.4 months with imlunestrant + abemaciclib and 5.5 months with imlunestrant alone (HR 0.57 *p* < 0.001)	N/R	Improvements in global health status/quality of life (GHS/QoL) and physical functioning with imlunestrant in patients with ESR1^mut^	Grade 3 or higher AEs were 17.1% with imlunestrant, 20.7% with standard therapy, and 48.6% with imlunestrant–abemaciclib	No ****
OLYMPIAD Olaparib (NCT02000622) [25]	2019	gBRCA1/2	Phase III, randomized, controlled, open-label study	152 HR+ pts	BRCA germline mutations	≤2 prior CT lines for MBC (prior treatment with anthracycline and taxane for EBC or MBC; ≤1 prior ET lines for MBC in HR + BC)	59.9%	Median PFS 8.0 months (HR 0.51)	Median OS 19.3 (Olaparib) vs. 17.1 months for TPC (HR 0.89, 95% CI 0.67–1.18)	HRQoL improved (EORTC QLQ-C30))	Grade 3+ AEs 36.6%. Discontinuation rate 5.4%	Yes
EMBRACA Talazoparib (NCT01945775) [26]	2019	gBRCA1/2	Phase III study	431 (HR+, 241)	BRCA germline mutation	≤3 prior CT lines for MBC for EBC or MBC; no limit on the number of prior ET lines in HR + BC	62.6%	Median PFS 8.6 months (HR 0.54 *p* < 0.001)	HR for OS was 0.848 (95% CI 0.670–1.073; *p* = 0.17).	Improvement and delay in time to definitive clinically meaningful deterioration (TTD)	Grade 3/4 AEs 25.5%. Discontinuation rate 5.9%	Yes

* In the CAPItello-291 trial, all patients were included regardless of mutation status, although the benefit was greater in the subgroup of patients with mutations in the PI3K/AKT/PTEN pathway. ** In the SERENA-2 trial, all patients were enrolled regardless of biomarker status. The study analyzed subgroups based on the presence of ESR1 mutations, but this was not an inclusion criterion. *** In the EMERALD trial, patients were included both with and without ESR1 mutations, although outcomes with elacestrant were superior in the ESR1-mutated group. ESR1 mutations were detected in 47.8% of enrolled patients. N/R: Not reported. **** Approval occurred prior to publication of this article.

EMBER-3, the phase 3 trial of the oral SERD Imlunestrant, was reported in 2024. The trial included patients with HR+ breast cancer that recurred or progressed during or after aromatase inhibitor therapy, but included patients who had received these with or without a CDK4/6 inhibitor. Participants were randomized in a 1:1:1 ratio to receive imlunestrant, standard endocrine monotherapy, or the combination of imlunestrant and abemaciclib. Among 256 patients with detectable ESR1 mutations in ctDNA, the median progression-free survival was 5.5 months with imlunestrant compared to 3.8 months with standard endocrine monotherapy. In a cohort of 426 patients comparing imlunestrant–abemaciclib with imlunestrant alone, the median PFS was 9.4 months and 5.5 months, respectively (HR 0.57; 95% CI, 0.44–0.73; *p* < 0.001). Grade 3 or higher adverse events were reported in 48.6% of patients receiving the imlunestrant–abemaciclib combination [22].

These findings are again consistent with other trials of oral SERDS and related agents, demonstrating that imlunestrant monotherapy extends PFS compared to standard therapy among patients with detectable *ESR1* mutations in ctDNA. In addition, the combination of imlunestrant and abemaciclib improves PFS compared to imlunestrant alone, regardless of *ESR1* mutation status. Consistent treatment effects were observed across subgroups, including those with prior visceral metastases, previous CDK4/6 inhibitor therapy, and *PI3K* pathway mutations. While these results suggest that more effective endocrine therapy and continuation or switching of CDK4/6 inhibitor has potential to improve outcomes, direct comparisons are unavailable, and prior treatment of enrolled participants is no longer typical of current practice (34.7% of participants in the imlunestrant–abemaciclib arm had received prior AI as monotherapy, and 64.7% of prior CDK4/6i use was palbociclib). This complicates the interpretation of these results and their application to patients with or without *ESR1* mutations [22].

Patient-reported outcomes in EMBER-3 demonstrated that patients with *ESR1m* experienced greater improvements in global health status/quality of life (GHS/QoL) and physical functioning with imlunestrant compared to standard-of-care, aligning with the observed efficacy outcomes [49].

Vepdegestrant is the first proteolysis-targeting chimera (PROTAC) to be evaluated in a phase 3 clinical trial, as well as the first PROTAC estrogen receptor degrader. In the phase 3 VERITAC-2 trial, vepdegestrant improved PFS compared to fulvestrant in patients with previously treated HR+/HER2-negative advanced breast cancer containing *ESR1* mutation [23]. The study enrolled 624 participants (including 270 with *ESR1* mutations), all of whom had received prior endocrine therapy and a CDK4/6 inhibitor. Among patients with detectable *ESR1* mutations, the median PFS was 5.0 months in the vepdegestrant group vs. 2.1 months in the fulvestrant group (HR 0.58; *p* < 0.001). In the overall population, however, median PFS was 3.8 months with vepdegestrant vs. 3.6 months with fulvestrant (HR, 0.83; *p* = 0.07). Grade 3 or higher adverse events occurred in 23.4% of patients treated with vepdegestrant and 17.6% of those treated with fulvestrant. Treatment discontinuation due to adverse events was reported in 2.9% of the vepdegestrant group and 0.7% of the fulvestrant group.

#### 2.2.3. Targeting BRCA Germline Mutations

In the phase III OlympiAD study, conducted prior to the routine adoption of CDK4/6i, olaparib significantly prolonged PFS compared to physician’s choice of non-platinum chemotherapy in patients with HER2-negative metastatic breast cancer with germline *BRCA* mutations (*gBRCAm*), without an improvement in OS. In the extended final analysis, the median OS was longer for olaparib in the first-line post-endocrine setting compared to its use in later lines [25,26]. Gelmon and colleagues evaluated the use of olaparib in real-world settings in the LUCY trial, with efficacy and safety results consistent with those found in OLYMPIAD. PFS was also evaluated based on prior exposure to a CDK4/6 inhibitor (yes vs. no, post hoc analysis). The median PFS was 7.95 months (*n* = 25) in those with prior CDK4/6i exposure and 8.34 months (*n* = 106) in those without. No formal statistical comparison was conducted in this subgroup [50].

The phase 3 EMBRACA trial similarly included patients with advanced *gBRCAm* breast cancer to receive talazoparib versus physician’s choice therapy. PFS was improved (HR 0.54, *p* < 0.0001) by 3 months. No benefit was observed in OS [25]. Savill et al. evaluated talazoparib in a real-world metastatic setting in 84 patients (35.7% with HR+, HER2-negative). Talazoparib was used as second-line therapy in 40.5% of the patients, and 89.7% had received a CDK4/6 inhibitor as first-line treatment. They observed a median progression-free survival of 8.7 months and an overall response rate of 63.1%, consistent with the findings of EMBRACA [51].

PAMBRACA, a real-world Italian study presented at ASCO 2025, showed that, following progression on CDK4/6 inhibitors plus endocrine therapy, patients with germline BRCA1 or BRCA2 mutations experienced a longer real-world progression-free survival (rwPFS) with PARPi compared to those receiving other treatment options such as single-agent chemotherapy, hormonal therapy (with or without everolimus), or combination chemotherapy. Moreover, early administration of PARP inhibitors following CDK4/6 inhibitor therapy was associated with longer median rwPFS compared with later use (13 vs. 6 months; aHR 2.81, 95% CI 1.15–6.90, *p* = 0.024) [52].

### 2.3. Second Line Irrespective of a Biomarker

#### Targeted Therapy

The combination of the mTOR inhibitor everolimus with the aromatase inhibitor exemestane was evaluated in the phase III BOLERO-2 randomized trial. It demonstrated an improvement in PFS with an absolute gain of 6.5 months, but it did not show a benefit in overall survival. Questions have been raised about the role of informative censoring on the results observed. However, a critical point to highlight is that the design and results of the BOLERO-2 trial predated the development of CDK4/6i. In a real-world analysis, patients with prior CDK4/6 inhibitor use had a lower median PFS of 3.8 months compared to 5.4 months for patients without prior CDK4/6 inhibitor use, with an HR of 1.46 (95% CI: 1.08–1.97, p = 0.013). Overall survival between the groups was not significantly different [53,54].

Correlative studies have been conducted to characterize the outcome of various subsets in BOLERO-2, including an analysis of PAM50 intrinsic subtype [54]. Independent of treatment arm, patients with luminal subtypes had a longer median PFS compared to those with non-luminal disease (6.7 vs. 5.2 months; adjusted HR 0.66; *p* = 0.020), indicating a better prognosis. Similarly, HER2-enriched tumors were associated with a shorter PFS than non–HER2-enriched tumors (5.2 vs. 6.2 months; HR 1.53; *p* = 0.019). These differences were observed regardless of everolimus exposure, suggesting that intrinsic subtype may reflect overall prognosis rather than differential treatment effect.

A secondary analysis of somatic mutations in the BOLERO-2 trial was conducted in 227 participants with available tumor material. Variants in 182 cancer-related genes were assessed using next-generation sequencing (NGS). The benefit of everolimus over placebo was maintained across subgroups defined by any of the nine genes with a mutation rate >10% (e.g., *PIK3CA, FGFR1, CCND1*). An exploratory analysis of baseline *ESR1* mutations (Y537S and D538G) using cfDNA was also performed. These mutations were detected in approximately 29% of patients and were associated with worse overall survival. Importantly, while ESR1 mutations are known to confer resistance to aromatase inhibitors such as exemestane, patients with the D538G mutation still derived progression-free survival benefit from the addition of everolimus. This highlights the importance of considering ESR1 mutational status when interpreting outcomes with different treatment backbones [55].

PrE0102 evaluated the combination of everolimus plus fulvestrant, compared to fulvestrant plus placebo, in 131 postmenopausal women with HR+/HER2-negative ABC resistant to aromatase inhibitors [56,57]. The addition of everolimus significantly improved median PFS from 5.1 months to 10.3 months (HR = 0.61; 95% CI: 0.40–0.92; *p* = 0.02), meeting the study’s primary endpoint. While objective response rates were similar between groups (18.2% vs. 12.3%; *p* = 0.47), the clinical benefit rate was significantly higher in the everolimus group (63.6% vs. 41.5%; *p* = 0.01). Of note, prior CDK4/6 inhibitor therapy use was negligible in this study (0% vs. 3%), but the results provide useful information about the potential for this combination in AI resistant disease.

The phase II MANTA trial evaluated the efficacy of fulvestrant plus vistusertib (a now-discontinued investigational inhibitor of mTOR) versus fulvestrant plus everolimus and fulvestrant alone in participants not pretreated with CDK4/6 inhibitors. In this study, median PFS was 5.4 months with fulvestrant alone, 7.6 months with fulvestrant plus daily vitusertib, and 12.3 months with fulvestrant plus everolimus. The trial did not meet its primary endpoint for vistusertib; however, the results provided an additional estimate of the activity of the fulvestrant plus everolimus arm [58].

Vasseur et al. conducted a prospective study to evaluate the efficacy of fulvestrant combined with everolimus previously treated with CDK4/6 inhibitors. The study also assessed changes in circulating tumor DNA during treatment. Among the 57 patients included, the median PFS was 6.8 months, and the median OS was 38.2 months. Baseline ctDNA detection and the presence of *PIK3CA* mutations were independently associated with worse PFS and OS. A decrease in ctDNA levels between baseline and weeks 3–5 was associated with better PFS and OS, suggesting that early ctDNA decline may serve as a marker of treatment response. However, discordant cases were observed, indicating that ctDNA may originate from tumor subclones that do not fully capture the overall tumor evolution under therapy, or that different methods or timing of measurement may be necessary [59].

Another agent under investigation in a biomarker-unselected fashion is gedatolisib, evaluated in the VIKTORIA-01 phase III trial in combination with fulvestrant, with or without palbociclib, for HR+/HER2− advanced breast cancer previously treated with a CDK4/6 inhibitor. Gedatolisib is an intravenous pan-PI3K/mTORC1/2 inhibitor, which may prevent cross-activation between pathway nodes (a resistance mechanism decribed with selective PI3Kα, AKT, or mTOR inhibition) [60,61]. A phase Ib study in 103 women with HR+/HER2– ABC evaluated gedatolisib plus palbociclib and endocrine therapy (letrozole or fulvestrant). The median PFS was 12.9 months, supporting the hypothesis that CDK4/6 inhibitor resistance may be overcome through PI3K/mTOR pathway inhibition, or that this pathway remains relevant in CDK4/6i-resistant disease [62].

Clinical data from the PIK3CA wild-type cohort of the VIKTORIA-01 trial reported via press release on 29 July 2025 showed that the combination of gedatolisib + fulvestrant + palbociclib produced a median PFS of 9.3 months versus 2.0 months with fulvestrant alone, with a HR of 0.24. In the doublet regimen (gedatolisib + fulvestrant), the mPFS was 7.4 months compared to 2.0 months with fulvestrant, with an HR of 0.33. While initial results are encouraging, further safety, efficacy, and enrollment details will be critical to define the role of this triplet in the current landscape. Stratification by endocrine sensitivity or prior therapy duration may also help contextualize the observed benefit, given the short PFS in the control arm [60].

Table 4 provides a summary of clinical trials evaluating non–biomarker-guided treatment options and their corresponding clinical outcomes.

### 2.4. CDK4/6 Inhibitor Maintenance Strategies

For patients whose disease progresses on a first-line CDK4/6 inhibitor combined with endocrine therapy and who do not have detectable actionable mutations, several studies suggest that including a CDK4/6 inhibitor in the second line may be a useful strategy. In the phase II MAINTAIN trial, the addition of ribociclib to endocrine therapy in patients who had previously received a CDK inhibitor (86.5% palbociclib) resulted in a statistically significant improvement in PFS (5.3 vs. 2.8 months, *p* = 0.006). However, no benefit was observed in patients who received prior ET+CDK4/6i for more than 12 months (HR, 0.76; 95% CI, 0.47–1.24). While an exploratory analysis of circulating tumor DNA suggested that the presence of an *ESR1* or *PIK3CA* mutation negated any benefit from adding ribociclib, these were small and post hoc analyses [63]. In contrast, the phase II PACE trial showed that continuing palbociclib (in combination with fulvestrant) beyond progression on palbociclib and an AI did not improve PFS compared to fulvestrant alone [64].

PALMIRA was a phase II study that enrolled 198 participants with HR+/HER2-negative ABC who experienced disease progression after first-line treatment with palbociclib plus endocrine therapy (AI or fulvestrant) [65]. Eligible patients had either derived clinical benefit from the prior regimen (response or stable disease ≥24 weeks) or had progressed on palbociclib-based therapy in the adjuvant setting. Participants were randomized 2:1 to receive palbociclib rechallenge plus second-line ET (fulvestrant or letrozole) or second-line ET alone. The median investigator-assessed PFS was 4.9 months with palbociclib plus ET versus 3.6 months with ET alone (HR 0.84; *p* = 0.149). While the primary endpoint of PFS was not improved with palbociclib rechallenge, the 6-month clinical benefit rate was significantly higher in the combination arm (41.9% vs. 27.4%; *p* = 0.044), suggesting that a subset of patients may benefit from continued CDK4/6 inhibition in the second-line setting.

The phase III postMONARCH trial was designed to address the uncertainty resulting from these Phase 2 trials and observational data [65,66,67]. postMONARCH randomized patients with disease progression on CDK4/6i and endocrine therapy to abemaciclib plus fulvestrant or placebo plus fulvestrant and met its primary endpoint of investigator-assessed PFS. The majority of participants were treated with palbociclib prior to enrolment (*n* = 217; 59%), which presents a limitation given the current first-line treatment landscape, and direct comparisons between different CDK4/6 inhibitors in this specific setting are not available. Median PFS was 6.0 months (95% CI, 5.6–8.6) with abemaciclib plus fulvestrant vs. 5.3 months (95% CI, 3.7–5.6) (HR 0.73; 95% CI, 0.57–0.95; *p* = 0.017). A PFS benefit with abemaciclib plus fulvestrant was observed regardless of *ESR1* or *PIK3CA* mutation status [62]. Greater benefit was seen in those without visceral metastases (HR 0.53), while those with visceral disease had limited benefit (HR 0.87). These findings highlight the potential importance of patient stratification when evaluating the efficacy of continued CDK4/6 inhibition in this setting.

Table 5 provides a summary of four studies investigating CDK4/6 inhibitor maintenance strategies following progression on first-line therapy, outlining their study designs and reported outcomes. EMBER-3 was included in Table 3.

### 2.5. ”1.5. Line” Therapy

The term “line 1.5” refers to a therapeutic intervention that occurs after the initiation of first-line treatment but before clinical progression that would warrant standard second-line therapy, as evaluated in the trials described above. In the SERENA-6 and PADA-1 trials, the detection of arising *ESR1* mutations in ctDNA in the absence of clinical progression during first-line therapy prompted a switch in endocrine therapy. This strategy is based on the identification of molecular resistance, with the hypothesis that early intervention may delay clinical progression and prolong progression-free survival [24,36]. Implementing this approach requires regular biomarker monitoring, such as *ESR1* detection in circulating tumor DNA, along with the availability of effective therapies to counteract the identified resistance pathways.

The PADA-1 trial was the first study in breast cancer to evaluate this strategy, and provided proof of concept for this approach [36]. This trial enrolled patients with HR+/HER2-negative advanced breast cancer initiating first-line AI therapy with palbociclib. Patients underwent ctDNA monitoring every two months for *ESR1* mutations using a targeted PCR assay, and upon mutation detection without clinical progression, they were randomized to either continue AI plus palbociclib or switch to fulvestrant plus palbociclib. The median time before the detection of ESR1 mutation in the study was 18 months. The trial demonstrated that an early switch to fulvestrant plus palbociclib upon *ESR1* mutation detection doubled PFS compared to standard treatment (no treatment change) (11.9 months vs. 5.7 months HR, 0.61; *p* = 0.004). In a multivariable analysis, factors associated with a higher incidence of synchronous progressive disease in patients with ESR1 mutations were compared to those without disease progression despite containing ESR1 mutations. These included tumor grade of 3 (OR, 2.56) and a metastasis-free interval of more than 3 years (OR, 6.61) [66].

In the latest update of PADA-1, the kinetics of *bESR1mut* (measured using digital droplet PCR at baseline, 1 month, and every 2 months thereafter) were assessed, identifying baseline factors associated with *bESR1mut* emergence. Factors independently associated with an increased likelihood of rising *bESR1mut* included the presence of bone metastases, higher estrogen receptor expression, younger age, and elevated baseline lactate dehydrogenase (LDH) levels [67,68].

Based on the results of PADA-1, SERENA-6 was designed as a phase III, randomized, double-blind trial in patients with hormone receptor-positive HR+/HER2-negative ABC receiving a first-line aromatase inhibitor in combination with a CDK4/6 inhibitor [24,36]. Patients receiving 1L therapy were enrolled and monitored patients for emerging *ESR1* mutations through serial assessments of ctDNA every 2 to 3 months using the Guardant360 NGS platform. All participants had received at least 6 months of first-line treatment with an AI in combination with a CDK4/6 inhibitor. Patients who developed an *ESR1* mutation but were deemed not to have clinical or radiographic disease progression were randomly assigned 1:1 to switch to camizestrant while continuing the CDK4/6 inhibitor (with placebo replacing the AI) or to continue their current regimen of an AI plus CDK4/6 inhibitor (with camizestrant placebo). Among 3256 patients tested for *ESR1* mutations, 315 met eligibility criteria and were randomized, 157 to the camizestrant arm and 158 to the AI arm. After a median follow-up of 12.6 months, the median PFS was 16.0 months in the camizestrant group compared to 9.2 months in the AI group (HR, 0.44; *p* < 0.0001). As a secondary outcome, PFS2 was evaluated, though this did not meet the threshold for statistical significance in the interim analysis (HR 0.52, 95% CI; *p* = 0.0038). At 12 months after random assignment, 85.4% of patients in the camizestrant arm had not experienced progression on second-line therapy compared with 74.4% of patients in the AI arm. Camizestrant was well-tolerated, with only 1.3% of patients discontinuing because of adverse events. Grade ≥ 3 adverse events (mostly attributable to the partner CDK4/6i) occurred more frequently in the camizestrant arm compared with the AI arm (60.0% vs. 45.8%). Notably, patient-reported median time to deterioration in global health status and quality of life was 23.0 months in the camizestrant group versus 6.4 months in the AI group [24]. The results of SERENA-6 solidify the notion that arising ESR1 mutations can be detected in ctDNA, and that endocrine therapy switch upon molecular progression can extend disease control. The longer-term impacts, including on overall survival, remain to be seen, and the contribution of this surveillance and intervention approach vs. switching to a camizestrant backbone while maintaining CDK4/6i at clinical disease progression has not been evaluated.

### 2.6. Prognostic Biomarkers: Circulating Tumor-DNA Dynamics (ctDNA)

In addition to the duration of prior CDK4/6 inhibitor therapy and the presence of visceral metastases, additional prognostic biomarkers may help guide treatment decisions for patients receiving endocrine-based strategies. While limited data have been reported in the post-CDK setting, a growing body of evidence for ctDNA fraction as a prognostic biomarker is emerging. Baseline ctDNA levels and changes on treatment have been assessed in several CDK4/6i studies. Pretreatment plasma samples from 459 PALOMA-3 participants were analyzed for mutations across 17 genes. Patients with a high circulating tumor DNA fraction had shorter PFS in both arms. In multivariable analysis, high ctDNA fraction, *TP53* mutations, and *FGFR1* amplification were all independently associated with worse PFS [69]. Chia et al. analyzed patients from MONALEESA-3 trial and reported associations between longitudinal changes in ctDNA using next-generation sequencing (NGS) and outcomes. Notably, ctDNA detection and percentage change remained significantly associated with PFS and OS even after adjusting for changes in tumor size. These findings provide proof of concept that longitudinal ctDNA analysis is a potential method for monitoring disease progression and may serve as a potential surrogate marker of treatment efficacy in HR+/HER2− advanced breast cancer [70,71].

Martínez-Sáez et al. analyzed the levels of ctDNA as a predictive marker for the response to anticancer therapies, including CDK4/6 inhibitors [72]. In this study, the mean variant allele fraction ratio (mVAFR), assessed using a standardized multigene panel was significantly associated with progression-free survival. In contrast, baseline variant allele fraction (VAF), on-treatment VAF, and absolute changes in VAF did not correlate with PFS.

A recent report from our institutional LIBERATE cohort study, which included 51 patients with HR+/HER2− MBC receiving standard ET and CDK4/6i evaluated ctDNA detection and dynamics using a high-sensitivity tumor-informed assay. Detection sensitivity was high compared to those reported with standard genotyping assays (91% baseline, 70% all timepoints), and associations between higher baseline estimated variant allele fractions, liver metastases, and shorter time to treatment failure and OS were observed. The use of a high-sensitivity assay (limit of detection ~0.001%, vs. ~0.1–0.3% for genotyping assays) permitted a more specific ascertainment and characterization of complete molecular response, defined as ctDNA clearance: the lowest estimated variant allele fraction (eVAF) in a sample called as having ctDNA detected was 0.0008%. ctDNA clearance was observed in 11 (28%) of the 39 patients who had detectable ctDNA at baseline, and the median time to its first occurrence was 5.7 months. ctDNA clearance with this assay was associated with improved TTF (HR 0.07) and OS (HR 0.07) [73]. Further, among participants where treatment failure occurred, rising ctDNA was observed prior to clinical progression.

Evidence for the association between ctDNA features, ctDNA kinetics and clinical outcomes in second-line therapy (post-CDK4/6i and AI), was provided from the phase I/II of GSK525762 combined with fulvestrant. Here, baseline ctDNA detection and the presence of copy-number alterations (CNA) were correlated with PFS, and persistence of these after the first cycle of treatment was associated with worse outcome [74].

### 2.7. Ongoing Phase 3 Clinical Trials in Second Line

While the second-line post-CDK4/6i standard-of-care remains incompletely defined based on completed trials, multiple phase 3 studies continue in this setting (Table 6), and are likely to further influence future clinical management. Agents and combinations under investigation include next-generation endocrine therapies, novel PI3K-targeted agents, and other emerging classes such as KAT6 inhibitors.

Following the evidence and approvals for PIK3CA and AKT inhibitors in the second line, described above, several next generation agents and combinations are being evaluated in phase 3 trials in this setting. Inavolisib is a selective PI3Kα inhibitor, which has been approved in combination with fulvestrant and palbociclib in patients recurring on or shortly after adjuvant therapy with an aromatase inhibitor, on the basis of the INAVO120 trial. Extending the evaluation of this into the post-CDK4/6i setting, INAVO-121 is evaluating inavolisib + fulvestrant vs. alpelisib and fulvestrant as a second-line treatment in participants with PIK3CA mutations after progression on a CDK4/6i and AI regimen [75]. Also having recently entered phase 3 evaluation, RLY-2608 is a mutant-selective PI3Kα inhibitor being tested in combination with fulvestrant compared to capivasertib and fulvestrant in patients with PIK3CA-activating mutations [76]. STX-478 is an allosteric PIK3CAα inhibitor designed to selectively target tumors harboring prevalent helical- and kinase-domain mutations in the PIK3CAα subunit. In preclinical models, STX-478 demonstrated potent antitumor activity in human xenografts without inducing the metabolic dysfunction commonly associated with alpelisib. Currently under investigation in phase 1/2 clinical trials for the treatment of breast cancer, STX-478 aims to achieve improved selectivity over existing PIK3CAα inhibitors, with the goal of enhancing efficacy while minimizing toxicity. Based on these results, a phase 3 trial is currently being planned to further evaluate its clinical potential [77].

Several additional Phase 3 trials are evaluating novel endocrine therapies in combination with continued CDK4/6i, or other existing targeted therapies. For example, evERA BC (NCT05306340) is a global, randomized phase III study comparing the oral SERD giredestrant plus everolimus versus exemestane plus everolimus in patients with HR+/HER2− ABC previously treated with endocrine therapy and a CDK4/6 inhibitor (*n* = 320) [78]. The study’s co-primary endpoints are PFS in both the overall and ESR1-mutant populations

Another novel target being evaluated in phase 3, based on recently published promising phase 2 data, is PF-07248144, a first-in-class catalytic inhibitor of KAT6, a lysine acetyltransferase involved in histone modification. Efficacy data have been published (*n* = 43), showing an ORR of 30.2% and a median PFS of 10.7 months (95% CI: 5.3–not evaluable) [79]. Updated results from the phase 1 trial presented at ASCO 2025 confirmed the combination dose with fulvestrant of 5 mg once daily, which had an acceptable safety profile and promising antitumor activity. In the monotherapy cohort, patients had received a median of 5 prior lines of therapy. All participants in both groups had prior endocrine therapy and exposure to a CDK4/6 inhibitor [79]. A phase 3 trial in the post-CDK4/6i setting will evaluate clinical efficacy compared to everolimus in combination with either exemestane or fulvestrant [80].

Beyond these studies of second-line endocrine ± targeted therapy, several phase 3 trials of antibody–drug conjugates are now enrolling patients in the immediate post–AI–CDK4/6i setting, a population previously excluded or requiring multiple lines of endocrine therapy or early progression. Ongoing trials include TroFuse-010 (NCT06312176) and HERTHENA-Breast04.

**Table 6:** Investigational phase III trials evaluating drug options after progression on first-line treatment.

Trial/Drug	Drug Mechanism of Action	Design	Patient Characteristics	Primary Endpoint	Secondary Endpoints
VIKTORIA-01: Gedatolisib in combination with fulvestrant, with or without palbociclib (NCT05501886) [61]	Pan-PI3K, mTORC1, and mTORC2 inhibitor	Phase III trial	HR+/HER2− ABC previously treated with a CDK4/6i	PFS	OS, DOR, TTR, CBR, QoL
TroFuse-010: sacituzumab tirumotecan (MK-2870) alone or in combination with pembrolizumab (NCT06312176) [81]	Trop2 antibody–drug conjugate (ADC)	Phase III	HR+/HER2− ABC with disease progression on one or more lines of ET with one in combination with a CDK4/i	PFS (sacituzumab tirumotecan versus TPC; pembrolizumab + sacituzumab tirumotecan versus TPC)	OS, ORR, PFS, DOR, QoL
INAVO-121: Inavolisib Plus Fulvestrant Compared With Alpelisib Plus Fulvestrant (NCT05646862) [75]	PI3Kα Inhibitor	Phase III	HR+/HER2− ABC with prior CDK4/6i in combination with ET	PFS	OS, ORR, BOR, DOR, CBR, TTCD
OPERA-01: Palazestrant as a single agent compared with SOC ET (NCT06016738) [82]	Oral complete ER antagonist (CERAN) and selective ER degrader (SERD)	Phase III	HR+/HER2− ABC with ≤2 prior ET (prior CDK4/6 inhibitors allowed) and ≤1 prior line of chemotherapy	PFS and Dose-Selection	OS
KATSIS-1: PF-07248144 in Combination With Fulvestrant (NCT07062965) [80]	Oral KAT6A and KAT6B inhibitor	Phase III	HR+/HER2− ABC with prior ET and CDK4/6i	PFS	OS, ORR, DOR, CBR, AEs
ReDiscover-2 Trial RLY-2608 + Fulvestrant compared with capivasertib plus fulvestrant (NCT06982521) [77]	Mutant-Selective Allosteric PI3Kα Inhibitor	Phase III	PIK3CA-mutant HR+/HER2− ABC with prior ET and CDK4/6i	PFS	OS, PFS (overall and kinase populations), DOR, CBR, AEs, PK, QoL
HERTHENA-Breast04/Patritumab deruxtecan compared with investigator’s choice of treatment (NCT07060807) [83]	HER3 directed ADC	Phase III	HR+/HER2− ABC with 1 prior ET and CDK4/6i	PFS, OS	ORR, DOR, TTD, QoL, safety, tolerability
evERA trial Giredestrant + everolimus compared with the Physician’s choice of Endocrine Therapy plus Everolimus (NCT05306340) [78]	Oral SERD	Phase III	HR+/HER2− ABC with prior CDK4/6i and ET	PFS	OS, ORR, DOR, CBR, PROs, safety and AEs
EPIK-B5 trial Alpelisib plus fulvestrant compared with placebo plus fulvestrant (NCT05038735) [84]	PI3Kα Inhibitor	Phase III	HR+/HER2− PIK3CA-mutated ABC progressing on/after an AI with a CDK4/6i	PFS	OS, ORR, DOR, CBR, TTR, PFS (by PIK3CA ctDNA), PFS on next-line treatment, TTD, QoL, safety, tolerability

Abbreviations: OS, overall survival; ORR, overall response rate; PFS, progression-free survival; DOR, duration of response; BOR, best overall response; CBR, clinical benefit rate; TTR, time to response; TTCD, time to confirmed deterioration; TTD, time to deterioration; PROs, patient-reported outcomes; QoL, quality of life; AEs, adverse events; PK, pharmacokinetics.

### 2.8. Moving Targets—Emerging Early Line Straties

While these second-line studies are ongoing, there remains significant investigational activity in the first-line setting. Trials in the first-line (1 L) and “1.5-line” (1.5 L) settings are expected to influence second-line approaches and may even reshape the interpretation of existing second-line trials. A key example is INAVO120: despite low crossover rates (<10%), the survival advantage underscores the impact of PIK3CAα inhibition. While this study design does not unequivocally support its exclusive use in the first-line setting, it highlights the complexity of having multiple targeted therapies available in combination with the same endocrine partner (fulvestrant). In addition, the results raise uncertainties about the optimal CDK4/6 inhibitor partners and the role of fulvestrant-based regimens in patients who have not received an aromatase inhibitor (e.g., those treated with adjuvant tamoxifen). The increasing use of adjuvant CDK4/6 inhibitors may further shape this clinical scenario.

In addition, as previously discussed, first-line treatment selection directly influences second-line therapeutic choices. This is especially relevant in the interpretation of trials comparing oral SERDs versus AIs, as well as those evaluating combinations with CDK4/6 inhibitors. Several ongoing studies will complicate this therapeutic landscape. For instance, SERENA-4 and persevERA are phase III trials being conducted in the first-line setting, comparing oral SERD (camizestrant and giredestrant, respectively) plus palbociclib to AI plus palbociclib [85,86].

## 3. Discussion: Optimizing Treatment in an Evolving Landscape

While first-line treatment for HR+/HER2-negative breast cancer with a CDK4/6 inhibitor and aromatase inhibitor is now well established as standard of care, the optimal approach to second-line therapy remains less clear. In part this reflects the parallel development of multiple strategies, during a time that outcomes for patients treated with standard endocrine therapy following CDK4/6i treatment failure were not initially understood. There is a growing appreciation that CDK4/6i-resistant disease is clinically and biologically heterogenous, and that individualized use of available second-line treatment—guided by tumor biomarkers and patient factors—is essential to maximize the potential of such strategies, while avoiding rapid and symptomatic disease progression.

The identification of predictive biomarkers and the use of molecular profiling are therefore essential to personalize post-CDK4/6 inhibitor (CDK4/6i) treatment strategies. Diagnostic tests with appropriate analytical performance should be employed. When seeking to detect *ESR1* mutations, testing should be performed on samples obtained after the development of resistance to endocrine therapy, in order to optimize the likelihood of mutation detection [87]. *ESR1* somatic mutations are associated with acquired resistance to ET, particularly in the metastatic setting, and are present in approximately 40% of patients with progressive HR+ metastatic breast cancer. In contrast, they are uncommon in de novo or relapsing disease [88]. *PIK3CA* mutations, on the other hand, are often truncal, and allow the use of targeted pathway inhibitors on the basis of either archival tissue or contemporaneous liquid biopsy samples [89].

A key question in clinical practice is the optimal timing for next-generation sequencing, as well as the preferred source for testing (tissue versus liquid biopsy). Until 2020, the European Society for Medical Oncology (ESMO) guidelines did not recommend routine NGS testing, instead favoring PCR for *PIK3CA* detection [8]. However, recent updates have reclassified *ESR1* mutations as ESCAT Level IA (based on results from the EMERALD trial) and now recommend NGS testing of tumor or plasma samples for HR+/HER2− ABC as standard of care [90]. Furthermore, the SAFIR-02 trial demonstrated improved progression-free survival with the use of targeted therapies in patients harboring ESCAT Level I/II genomic alterations [91].

Additional clinical challenges include the fragmentation of patient populations and the absence of clear guidelines for treating patients with concurrent genomic alterations. For example, in patients with both *ESR1* and AKT pathway mutations, no established sequential treatment strategy exists, and no approved PIK3CA/AKT inhibitor regimen includes an oral-SERD backbone. Although the exact prevalence of concurrent PIK3CA and ESR1 mutations is not consistently reported across clinical trials, real-world evidence indicates that such co-alterations occur in approximately 4–17% of patients, depending on the line of therapy [92]. Notably, in EMERALD, 34.6% of patients had *PI3K* mutations, while the CAPItello-291 trial did not report ESR1 mutational data (no ctDNA analysis has been presented). It has been suggested that capivasertib may be preferable to elacestrant monotherapy in patients with high disease burden and early progression following CDK4/6 inhibitor therapy, given the modest PFS (4 months) observed with elacestrant in such scenarios [19,21].

Treatment decisions could incorporate not only molecular profiles but also patient-specific prognostic factors. In the SUMA-001 study, Waisberg et al. found that postmenopausal status (HR 0.65, *p* = 0.038) and primary tumor Ki67 > 30% (HR 2.37, *p* = 0.02) were associated with second-line PFS. An interaction between menopausal status and treatment selection was observed (*p* = 0.015), suggesting that endocrine sensitivity may vary according to physiological state, but may also reflect prior therapies used [93]. Among patients who had not received prior chemotherapy, postmenopausal patients had significantly longer PFS compared to premenopausal patients (8.7 vs. 4.3 months; *p* = 0.0006). Additionally, retrospective evidence indicates that *BRCA* mutations may impact CDK4/6 inhibitor efficacy in the first-line setting [94]; however, no data are available for the second-line context in this population.

Another pressing issue is that, although single-agent endocrine therapy is generally not favored in second-line treatment, many trials have continued to use fulvestrant monotherapy as the control arm. According to recent ASCO guidelines, patients with low disease burden, long duration of response (≥12 months) to CDK4/6 inhibitors, and absence of actionable mutations may still be appropriate candidates for endocrine monotherapy [95].

**Figure 1:**
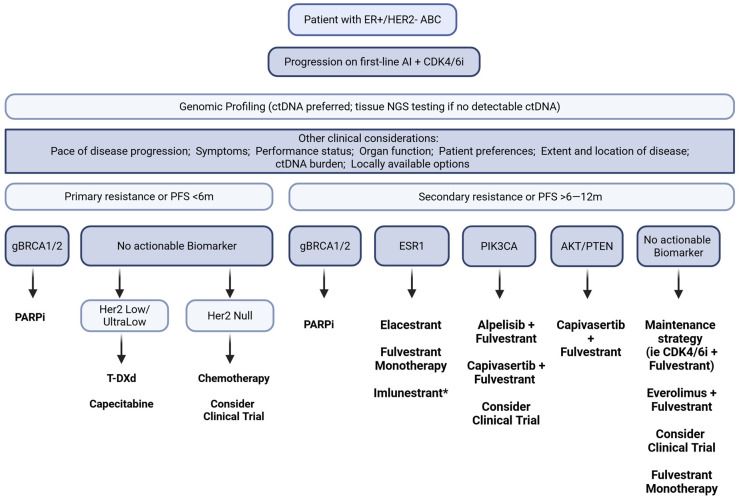
Flowchart of biomarker-guided treatment sequencing in patients with advanced HR+/HER2− breast cancer. The diagram illustrates therapeutic decision-making based on the presence or absence of key molecular alterations. * Imlunestrant was FDA approved on 25 September 2025, during the publication process of this article.

Although guidelines recommend three lines of endocrine therapy before chemotherapy, real-world data show that over 25% of patients receive chemotherapy as second-line treatment after CDK4/6i progression [12]. The rise of ADCs is reshaping post-CDK care and may challenge the routine application of endocrine-based therapy in this setting [96,97]. To remain relevant, ET-based regimens must be tolerable and provide a realistic likelihood of benefit for the individual patient. Determining endocrine suitability has therefore become critical, requiring both prognostic markers to identify who may derive meaningful benefit and predictive biomarkers to guide selection among oral SERDs, continued CDK4/6i, PI3K/AKT inhibitors, and ADCs. These strategies are not one-size-fits-all: careful individualization is needed to delay premature escalation to chemotherapy while preserving options that could still provide benefit. Adaptive strategies incorporating ctDNA monitoring may offer a path to treatment individualization and dynamic response assessment, but they require a shift in our therapeutic approach and robust clinical validation through prospective studies. Figure 1 shows the biomarker-driven sequencing framework that could be considered after CDK4/6 inhibitor progression.

Given the ongoing flux in clinical treatment standards, clinical trial participation remains an important option. Here, there is no singular profile for the ‘ideal’ clinical trial candidate, underscoring the need for thoughtful, patient-centered evaluation. To maximize the interpretability and impact of trial findings, it is essential that study designs reflect contemporary standards for both prior and post-protocol therapy, including crossover where appropriate.

## 4. Limitations of the Review

Many included trials are ongoing or have reported only preliminary findings, limiting the strength of conclusions. Several new agents lack global approval, and their role in real-world practice remains uncertain. Much of the available evidence comes from phase II trials, retrospective analyses, or subgroups, introducing bias and limiting generalizability. Differences in study populations (treatment line, prior endocrine exposure, duration of CDK4/6i use) further complicate comparisons. As a narrative review, article selection relied partly on author judgment without formal risk-of-bias assessment or quantitative synthesis, and negative or neutral studies may be underrepresented. Biomarker analyses are hampered by heterogeneity in platforms and detection methods, reducing reproducibility. Finally, the therapeutic landscape is rapidly evolving, and results from ongoing studies may soon alter recommendations; robust evidence on sequencing outside of clinical trials is still lacking.

## 5. Future Directions

Future research should prioritize biomarker-guided strategies to optimize sequencing in HR+/HER2− advanced breast cancer, and consider clinical factors associated with prognosis or treatment benefit. Circulating tumor DNA is a promising tool for dynamically guiding therapy and matching treatments to patients. Robust real-world evidence is also needed to define the role of therapies developed in an earlier treatment landscape.

## 6. Conclusions

Second-line treatment after CDK4/6i progression remains a major challenge given the range of options and difficulty predicting individual outcomes. Decisions should integrate patient preferences, tumor characteristics, prior therapy, and actionable biomarkers such as ESR1 and PIK3CA mutations, AKT pathway alterations, HER2-low/ultra-low status, and germline BRCA mutations. Other key factors include duration of prior CDK4/6i exposure, disease burden and sites, comorbidities, and clinical trial availability.

## Figures and Tables

**Table 1:** Approved first-line options for the treatment of advanced HR-positive, HER2-negative breast cancer.

Drug Combination	Trial	OS vs. ET Monotherapy	PFS	Trial Population
Palbociclib with letrozole	PALOMA-2 (NCT01740427) [8]	Not statistically significant	24.8 months	Postmenopausal women with HR+, HER2− advanced breast cancer
Ribociclib in combination with an aromatase inhibitor	MONALEESA-2 (NCT01958021) [15]	Significant improvement in OS with ribociclib combination; median OS not reached vs. 40.0 months	25.3 months	Postmenopausal women with HR+, HER2− advanced breast cancer
Ribociclib in combination with an aromatase inhibitor or tamoxifen	MONALEESA-7 (NCT02278120) [9]	Median OS not reached vs. 40.9 months with placebo.	23.8 months	Premenopausal women with HR+, HER2− advanced breast cancer
Abemaciclib in combination with an aromatase inhibitor	MONARCH 3 (NCT02246621) [11]	Median OS 66.8 months vs. 53.7 months. Not statistically significant (HR 0.84 *p* = 0.06)	28.2 months	Postmenopausal women with HR+, HER2− advanced breast cancer

**Table 2:** Actionable mutations and targeted therapies in HR+/HER2-negative metastatic breast cancer.

Mutation	Targeted Therapy	Clinical Trial
PIK3CA	Alpelisib + Fulvestrant	SOLAR-1 [17]
	Inavolisib + Palbociclib + Fulvestrant	INAVO 120 [18]
	Capivasertib + Fulvestrant	CAPItello-291 [19]
	Ipatasertib + Fulvestrant	FINER [20]
ESR1	Elacestrant	EMERALD [21]
	Imlunestrant	EMBER-3 [22]
	Vepdegestrant	VERITAC-2 [23]
	Camizestrant	SERENA-6 [24]
BRCA1/2	Olaparib,	OlympiAD [25]
	Talazoparib	EMBRACA [26]
AKT1	Capivasertib + Fulvestrant	CAPItello-291 [19]
	Ipatasertib + Fulvestrant	FINER [20]
PTEN	Capivasertib + Fulvestrant	CAPItello-291 [19]
	Ipatasertib + Fulvestrant	FINER [20]

**Table 4:** No Biomarker-Guided Treatment.

Drug	Year	Design	*n*	Patient Characteristics	Previous Treatment Allowed	ORR	PFS Benefit	OS Benefit	QoL Benefit	Adverse Events
BOLERO Everolimus (NCT00863655) [54]	2012	Phase III	724	HR+ ABC who had recurrence or progression while receiving AI	Nonsteroidal AI	Local assessment 9.5% central assessment 7%	Median PFS 10.6 months vs. 4.1 months, (HR, 0.36; 95% CI, 0.27 to 0.47; *p* < 0.001).	Median OS 31.0 months (HR = 0.89; 95% CI 0.73–1.10; log-rank *p* = 0.14). No benefit	Longer time to definitive deterioration (TDD)	Most common AEs stomatitis (8%), anemia (6%). SAEs 23%. Discontinuation rate 19%.
PrE0102 Everolimus plus fulvestrant (NCT01797120) [57]	2018	Phase II randomized, double-blind, placebo-controlled	131	Postmenopausal women with HR+/HER2− ABC	Prior CDK4/6 inhibitor therapy 3%	ORR were similar (18.2% vs. 12.3%; *p* = 0.47)	Median PFS 10.3 months vs. 5.1 months (HR, 0.61 [95% CI, 0.40 to 0.92]; *p* = 0.02),	Median OS 28.3 months (95% CI, 19.5 to 29.6 months) vs. 31.4 months (95% CI, 21.8 to NR). *p* = ns	N/R	Most common AEs: mucositis 53%, fatigue 42%, rash 38%, anemia 31%, diarrhea 23% and hyperglycemia 19%. No SAEs attributed to therapy. Discontinuation rate 39% (24% for treatment-related toxicity)
VIKTORIA-1 gedatolisib ± palbociclib plus fulvestrant (NCT05501886) [61]	2025	Phase III	392	Patients with HR+/HER2- PIK3CA wild type ABC	Progression during or after CDK4/6 inhibitor + AI	N/R	Median PFS 9.3 months (triplet) vs. 2 months (fulvestrant) (HR 0.24) and 7.4 Months (doublet) vs. 2 months (HR 0.33)	N/R	N/R	Most common AE was stomatitis; hyperglycemia in 26%. Discontinuation rate 4%

**Table 5:** CDK4/6 inhibitor maintenance strategies after progression on first-line treatment.

Trial	Year	Design	*n*	Patient Characteristics	Intervention	PFS Benefit
MAINTAIN (NCT02632045) [63]	2022	Phase II	119	HR+/HER2– ABC patients who progressed during ET and CDK4/6i	Randomization 1:1 to receive ribociclib plus fulvestrant vs. placebo plus fulvestrant	Median PFS 5.2 vs. 2.7 months (HR 0.57; 95% *p* = 0.006).
PACE (NCT0314728) [64]	2023	Phase II	220	HR+/HER2– ABC patients who progressed on previous AI plus CDK4/6i	Randomization 1:1 to fulvestrant, fulvestrant plus palbociclib, or fulvestrant plus palbociclib and avelumab	Median PFS 4.8 with fulvestrant vs. 4.6 months with fulvestrant plus palbociclib (HR 1.11 *p* = 0.62). Median PFS with fulvestrant plus palbociclib and avelumab 8.1 months (HR 0.75 *p* = 0.23)
PALMIRA (NCT03809988) [65]	2023	Phase II	198	HR+/HER2– ABC patients who progressed after first-line endocrine therapy (AI or fulvestrant) plus CDK4/6i and who had derived clinical benefit from the first-line treatment	Randomization 2:1 to palbociclib plus second-line ET (letrozole or fulvestrant, depending on prior ET) vs. second-line ET alone	Median PFS 4.2 vs. 3.6 months (HR 0.80 *p* = 0.206)
PostMONARCH (NCT05169567) [66]	2024	Phase III	273	HR+/HER2– ABC patients who progressed on prior AI plus CDK4/6i in the metastatic setting, or recurrence during/after adjuvant ET plus CDK4/6i	Randomization 1:1 to abemaciclib 150 mg twice daily vs. placebo, both in combination with fulvestrant	Median PFS 6.0 vs. 5.3 months (HR 0.73 *p* = 0.02)
EMBER-3 imlunes-trant (NCT04975308) [22]	2024	Phase III	874	HR+/HER2− ABC patients who recurred or progressed during or after AI therapy, administered alone or with a CDK4/6i.	Randomization 1:1:1 ratio to receive imlunestrant, standard endocrine monotherapy, or imlunestrant–abemaciclib.	In the comparison of imlunestrant–abemaciclib with imlunestrant median PFS was 9.4 vs. 5.5 months (HR 0.57 *p* < 0.001)

## Data Availability

No new data were created or analyzed in this study. Data sharing is not applicable to this article.
